# Supplementary material for: GNAS/PKA signaling promotes aberrant osteochondral differentiation of Gli1+ tendon sheath progenitors
Source: EMBO J. 2025 Sep 1;44(20):5890–917. doi: 10.1038/s44318-025-00553-7 (PMC12528478; doi:10.1038/s44318-025-00553-7)
Supplement: Supplementary file 1 — Appendix [file 44318_2025_553_MOESM1_ESM.pdf]

Appendix for GNAS/PKA signaling promotes aberrant osteochondral differentiation of Gli1+ tendon sheath progenitors

Table of contents

**Appendix Figure S1**	**2**
**Appendix Figure S2**	**3**
**Appendix Figure S3**	**4**
**Appendix Figure S4**	**6**
**Appendix Figure S5**	**7**
**Appendix Figure S6**	**8**
**Appendix Figure S7**	**9**
**Appendix Figure S8**	**10**
**Appendix Figure S9**	**12**
**Appendix Figure S10**	**14**

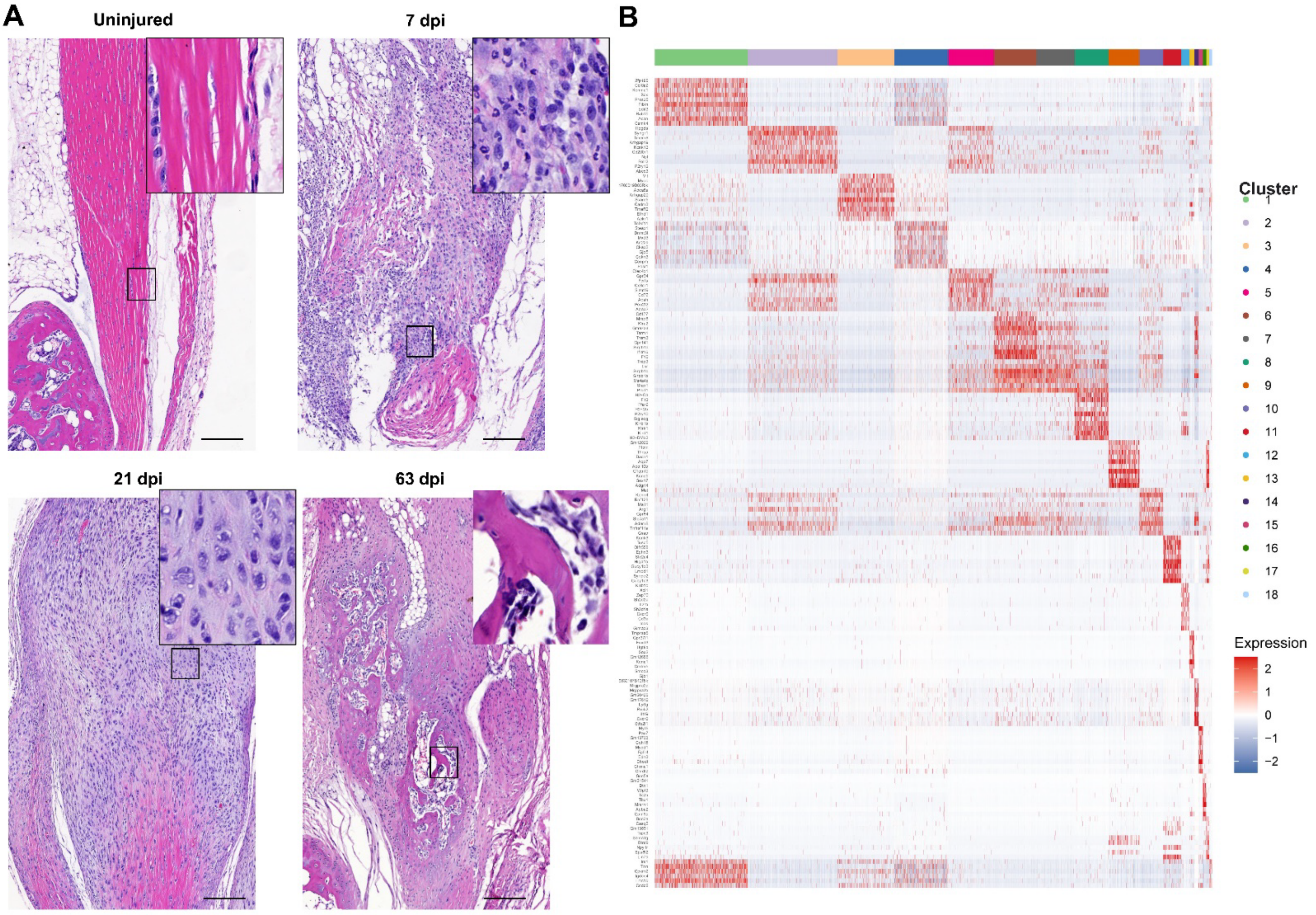

Appendix Figure S1. Validation of HO formation in injured mouse tendons. (**A**) Histology of uninjured tendons and injured tendons at 7, 21, and 63 dpi by HE staining. Scale bar, 200 µm. (**B**) Heatmap showing the top 10 differentially expressed genes (DEGs) for each cell cluster identified in traumatic HO lesions.

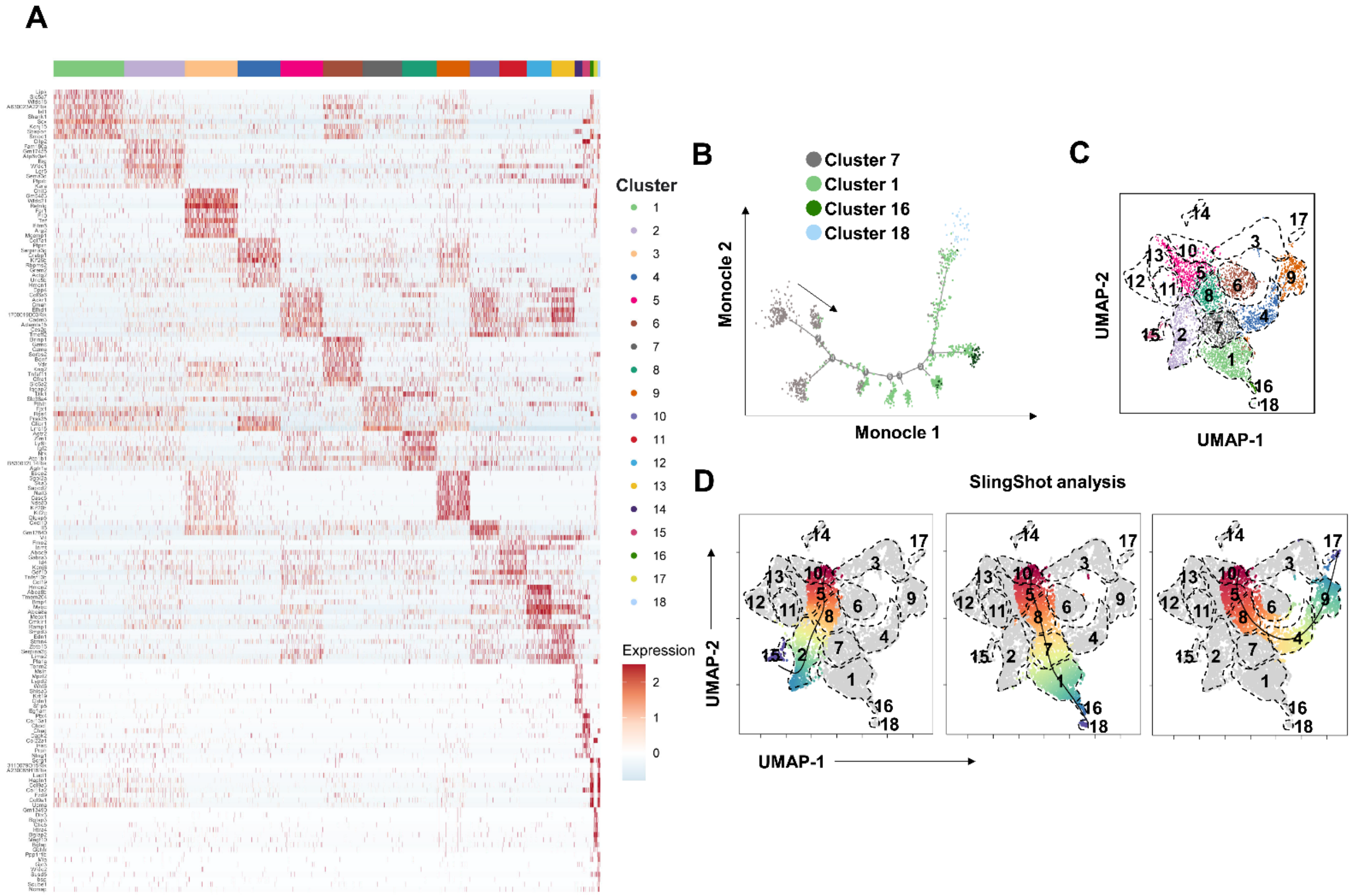

Appendix Figure S2. Trajectory analysis of tenogenic and osteochondrogenic differentiation by tendon stem/progenitor cells in heterotopic ossification (HO). (**A**) Heatmap displaying the top 10 DEGs for each cell cluster identified within mesenchymal and tendon-lineage cells isolated from traumatic HO lesions. (**B**) Monocle 2 analysis indicates differentiation of chondrogenic progenitors (clusters 1 and 7) into mature chondrocytes (clusters 16 and 18). (**C**) UMAP visualization depicting tendon stem/progenitor cells and their derivative cell populations. (**D**) Slingshot trajectory inference demonstrates differentiation of tendon stem/progenitor cells into mature chondrocytes, tenocytes, and osteogenic progenitors.

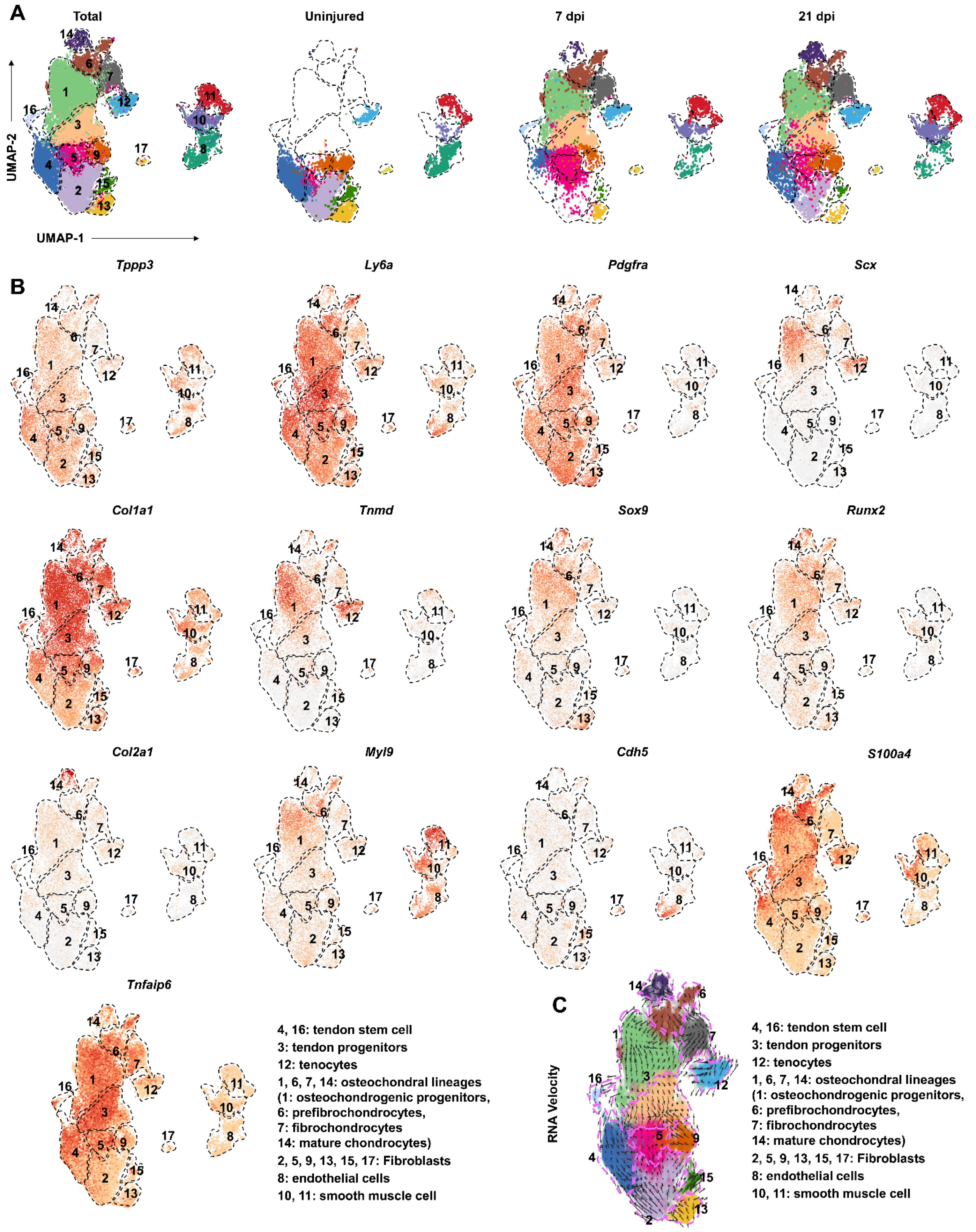

Appendix Figure S3. scRNA-seq analysis of tenogenic and osteochondrogenic differentiation in *Gli1*$^{-}$ cells. (**A**) UMAP visualization of *Gli1*$^{-}$ cells in uninjured tendons and injured tendons at post-injury time

points (specifically 7 and 21 dpi). (**B**) Feature plots showing expression of established markers for tendon stem/progenitor cells, vascular cells, smooth muscle cells, tenocytes, and chondrocytes. (**C**) RNA velocity analysis revealing tenogenic and osteochondrogenic differentiation trajectories of *Gli1*$^{-}$ tendon progenitor cells.

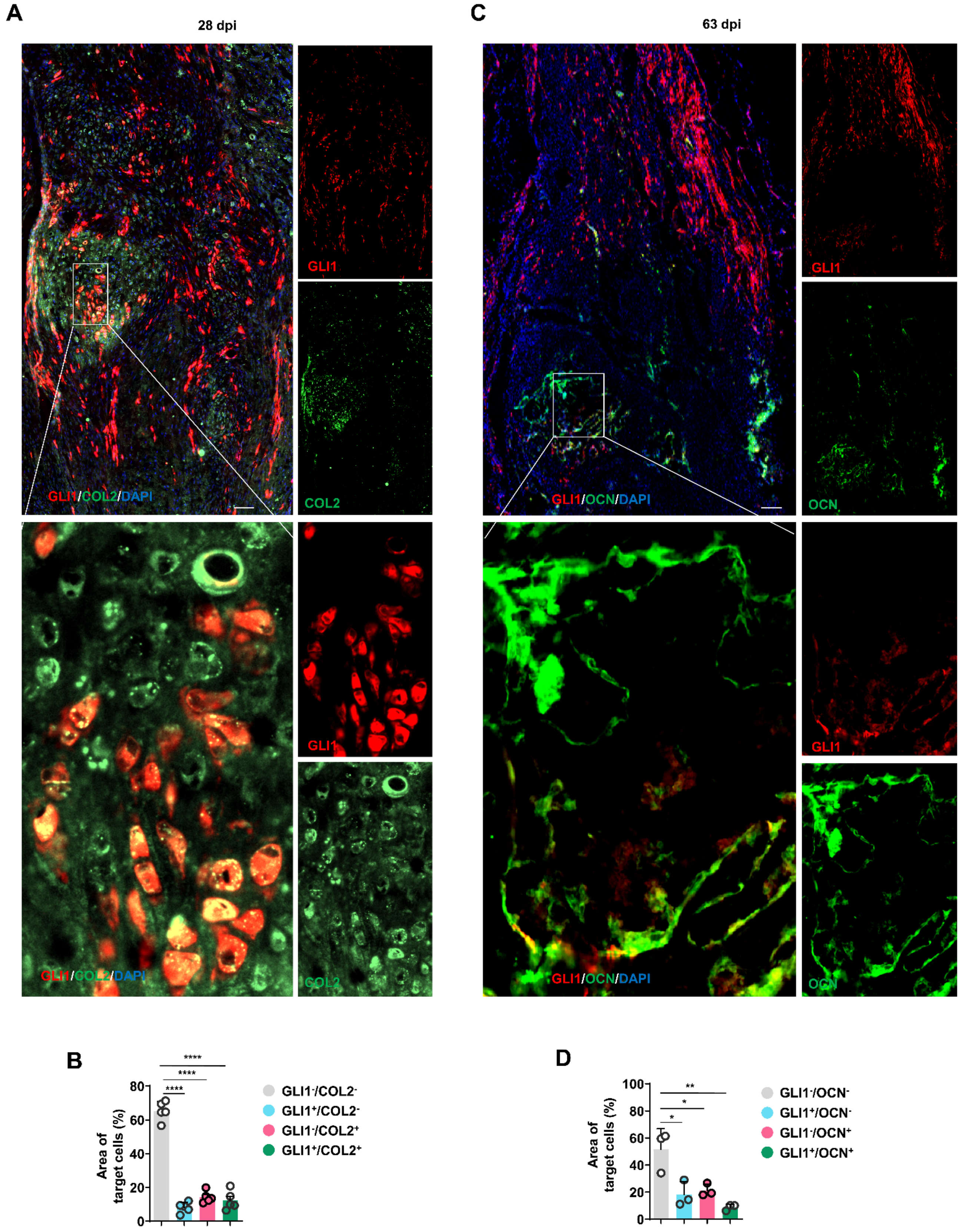

Appendix Figure S4. Chondrogenic and osteogenic differentiation potential of *Gli1*$^+$ tendon sheath progenitors. (**A**) Representative immunofluorescence images showing GLI1 and COL2 expression in tendon lesions at 21 dpi. Scale bar, 100μm. (**B**) Statistical analysis of frequency of GLI1$^-$/ COL2$^-$, GLI1$^+$/ COL2$^+$, GLI1$^-$/ COL2$^+$ and GLI1$^+$/ COL2$^-$ cells in injured site at 21 dpi (n=5 per group). Data is represented as the

mean ± SD. **** $p < 0.0001$. (**C**) Representative immunofluorescence images showing GLI1 and OCN expression in tendon lesions at 63 dpi. Scale bar, 100μm. (**D**) Statistical analysis of frequency of GLI1^{-}/ OCN^{-}, GLI1^{+}/ OCN^{+}, GLI1^{-}/ OCN^{+} and GLI1^{+}/ OCN^{-} cells in injured site at 63 dpi (n=3 per group). Data is represented as the mean ± SD. * $p < 0.05$, ** $p < 0.01$.

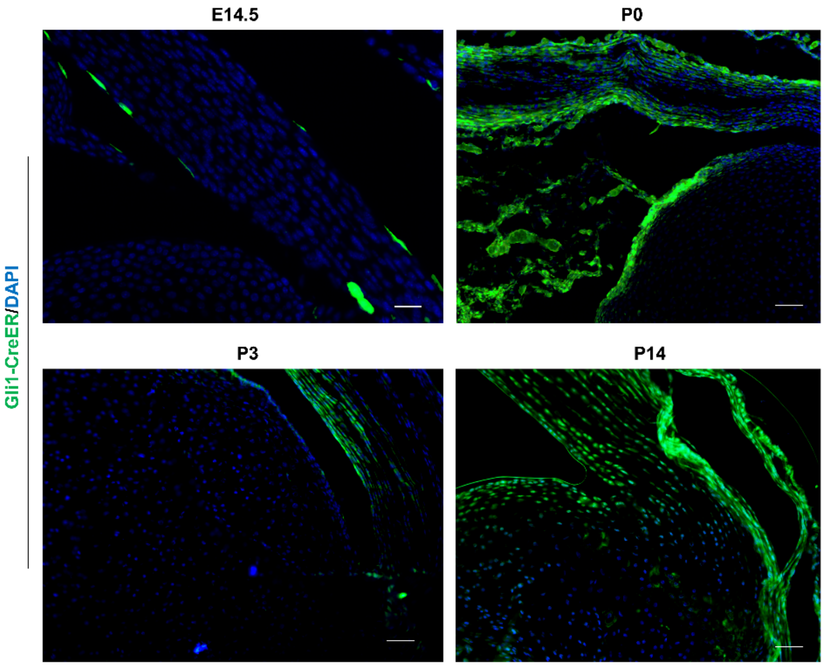

Appendix Figure S5. Lineage tracing of *Gli1*$^{+}$ tendon cells during developmental and postnatal stages. Scale bar, 50 μm.

Gli1-CreER^{T2}

ATAACTTCGTATAGCATACATTATACGAAGTTATTAGGGAGGAGATCAGCGCTTTCTCACTGGGATATTACAGTATAGAGGCT
GTTCATCAGGGCTCGTTGAAAAGCATCTTTGCATCTGAAGTCCACGTTCATAAGCGTTAACATCAAGGATTATGTGTCTCTT
ATATTTAAGAAGCTCTTAAGCATTCATGCATTAAAATACAGTTGATTCTTGGTGGAATCCTTTCTAAACCCCAGTTCTATTTAA
TTGATCACTTAAATGAGTGTCTGGGCCATTGTCTAAATGTTAGGAAGTATTTTAGTTGTGAATTGTTTTGATACGTGAGATGG
TTTGGTCGGGATCAGGAGCCATTACCTAAGATATTGGTAAATAACAAAGAAAACTGTGGTCTCCACGCCTACTTACTCTTTT
CTTCCCATTCTCCAGGGAAGTCCTTCCCCGGAACACAGAATTTCCACCTGGAAGTTGGCCTTATCATCCTCTCGGTGGTG
TTTGCAGTATGTCTTTTAGCTTGCATCCTTGGAGTTGCTCTCAGGAAGTTTAAGAGACGCAATCAAGAGCGCCTGAACCC
CAGAGACGTGGAGTATGGTACCATTGAAGGGCTCATCACCACCAATGTGGGAGACAGCACTCTAGCGGAACTACTAGAT
CACTCGTGTACATCAGGAAGTGGCTCCGGTCTTCCTTTCCTGGTACAGAGAACGGTGGCTCGCCAGATAACCCTGTTGG
AGTGTGTCGGGAAGGGCCGGTATGGAGAAGTATGGAGGGGCAGCTGGCAAGGCGAAAATGTCGCTGTGAAGATCTTCT
CCTCCCGAGACGAGAAGTCATGGTTCAGGGAGACGGAATTGTACAACACTGTGATGTTGAGGCATGAAAATATCTTAGGT
TTCATCGCTTCAGACATGACCTCCAGACACTCCAGTACCCAGCTGTGGCTCATCACACATTACCATGAAATGGGATCGTT
GTATGACTACCTTCAGCTCACTACTCTGGATACGGTTAGCTGCCTTCGGATTGTACTGTCCATAGCCAGCGGCCTTGCCC
ATTTGCACATAGAGATATTTGGGACCCAAGGGAAGTCCGCCATTGCCCATCGAGATCTGAAGAGCAAAAACATCCTGGTG
AAGAAGAATGGACAGTGCTGCATAGCAGATTTGGGCCTGGCAGTCATGCATTCCCAGAGCACAAACCAGCTTGATGTGG
GAAACAACCCCCGTGTGGGGACCAAGCGCTACATGGCTCCGGAAGTGCTCGATGAAACCATCCAAGTGGATTGCTTTGA
TTCTTATAAGAGGGTCGATATTTGGGCCTTTGGCCTTGTTCTGTGGGAAGTGGCCAGGCGAATGGTGAGCAATGGTATAG
TGGAAGATTACAAGCCACCATTCTATGATGTGGTTCCCAATGACCCAAGTTTTGAAGATATGAGGAAAGTTGTCTGTGTGG
ATCAACAGAGGCCAAACATACCTAACAGATGGTTCTCAGACCCGACATTAACTTCTCTGGCGAAGCTGATGAAAGAGTGC
TGGTATCAGAACCCATCCGCAAGACTCACAGCTCTACGTATCAAAAAGACTTTGACCAAAATCGATAATTCCCTAGACAAA
TTAAAAACTGACTGTTGACTGTAAGTCTGCAGAAATTGATGATCTATTAAACAATAAAGATGTCCACTAAAATGGAAGTTTTT
CCTGTCATACTTTGTTAAGAAGGGTGAGAACAGAGTACCTACATTTTGAATGGAAGGATTGGAGCTACGGGGGTGGGGGT
GGGGTGGGATTAGATAAATGCCTGCTCTTTACTGAAGGCTCTTTACTATTGCTTTATGATAATGTTTCATAGTTGGATATCAT
AATTTAAACAAGCAAAACCAAATTAAGGGCCAGCTCATTCCTCCCACTCATGATCTATAGATCTATAGATCTCTCGTGGGAT
CATTGTTTTTCTCTTGATTCCCACTTTGTGGTTCTAAGTACTGTGGTTTCCAAATGTGTCAGTTTCATAGCCTGAAGAACGA
GATCAGCAGCCTCTGTTCCACATACACTTCATTCTCAGTATTGTTTTGCCAAGTTCTAATTCCATCAGAAGCTTGCAGATCT
GCGACTCTAGAGGATCGACTGTGCCTTCTAGTTGCCAGCCATCTGTTGTTTGCCCCTCCCCCGTGCCTTCCTTGACCCT
GGAAGGTGCCACTCCCACTGTCCTTTCCTAATAAAATGAGGAAATTGCATCGCATTGTCTGAGTAGGTGTCATTCTATTCT
GGGGGGTGGGGTGGGGCAGGACAGCAAGGGGGAGGATTGGGAAGACAATAGCAGGCATGCTGGGGATGCGGTGGGC
TCTATGGCTGCGACTCTAGAGGATCATAATCAGCCATACCACATTTGTAGAGGTTTTACTTGCTTTAAAAAACGTTTAAACC
TCCCACACCTCCCCCTGAACCTGAAACATAAAATGAATGCAATTGTTGTTGTTAACTTGTTTATTGCAGCTTATAATGGTTAC
AAATAAAGCAATAGCATCACAAATTTCACAAATAAAGCATTTTTTTCACTGCATTCTAGTTGTGGTTTGTCCAAACTCATCAA
TGTATCTTATCATGTCTGGATCTGCGACTCTAGAGGATCATAATCAGCCATACCACATTTGTAGAGGTTTTACTTGCTTTAAA
AAACCTCCCACACCTCCCCCTGAACCTGAAACATAAAATGAATGCAATTGTTGTTGTTAACTTGTTTATTGCAGCTTATAAT
GGTTACAAATAAAGCAATAGCATCACAAATTTCACAAATAAAGCATTTTTTTCACTGCATTCTAGTTGTGGTTTGTCCAAACT
CATCAATGTATCTTATCATGTCTGGATCTGCGACTCTAGAGGATCATAATCAGCCATACCACATTTGTAGAGGTTTTACTTGC
TTTAAAAAACCTCCCACACCTCCCCCTGAACCTGAAACATAAAATGAATGCAATTGTTGTTGTTAACTTGTTTATTGCAGCT
TATAATGGTTACAAATAAAGCAATAGCATCACAAATTTCACAAATAAAGCATTTTTTTCACTGCATTCTAGTTGTGGTTTGTCC
AAACTCATCAATGTATCTTATCATGTCTGGATCCCCATCAAGCTGATAACATACGCTCTCCATCAAAACAA**AACGAAACA**AA
ACAAACTAGCAAAATAGGCTGTCCCCAGTGCAAGTGCAGGTGCCAGAACATTTCTCTGTTCCCATGGCTCCTTCTATTACT
AGATCAAGGGGTTTTGTGAGGGTTGCCCCAAGTCTGTGTGGGTTAAGGATCTGCGTAGCCAGAAGCATTCTTCCGACTT
GCTGAGCCAGAGCTGCTGGGCCATGGAAGGTCACTCTGGGTGAGGAAGACAAAGGCATGGCCTTTGGGATTGAAAAGG
AGAGTGATGTTTTGGGGAAAGTGAGTGATGTGACATGCATCCAGAGGAAGGGTGAAGGGGACGGATTCGCTGACTGCA
GGTCATCACAAATGGGTGTATGGGGCATGTGTGTTTCGAGAGAAGTAGGAACACTAGCTTGGTGTCTGTGTG**ATAACTTC**
GTATAGCATACATTATACGAAGTTAT

Gli1-CreER^{T2} ; Acvr1$^{R206H/+}$

ATAACTTCGTATAGCATACATTATACGAAGGGGACGGATTCGCTGACTGCAGGTCATCACAAATGGGTGTATGGGGCATGT
GTGTTTCGAGAGAAGTAGGAACACTAGCTTGGTGTCTGTGTG**ATAACTTCGTATAGCATACATTATACGAAGTTAT**

1bp~34bp and 3524bp~3590bp are loxP sites, 430bp~1628bp is WT CDS, 1629bp~3222bp is 5xStop

The yellow indicates the first loxP site; the green indicates the second loxP site; the red represents the deleted sequence.

Appendix Figure S6. Sanger sequencing verification of the loxP site and STOP cassette sequences in *Gli1-CreER^{T2}* and *Gli1-CreER^{T2}*; *Acvr1$^{R206H/+}$* mice.

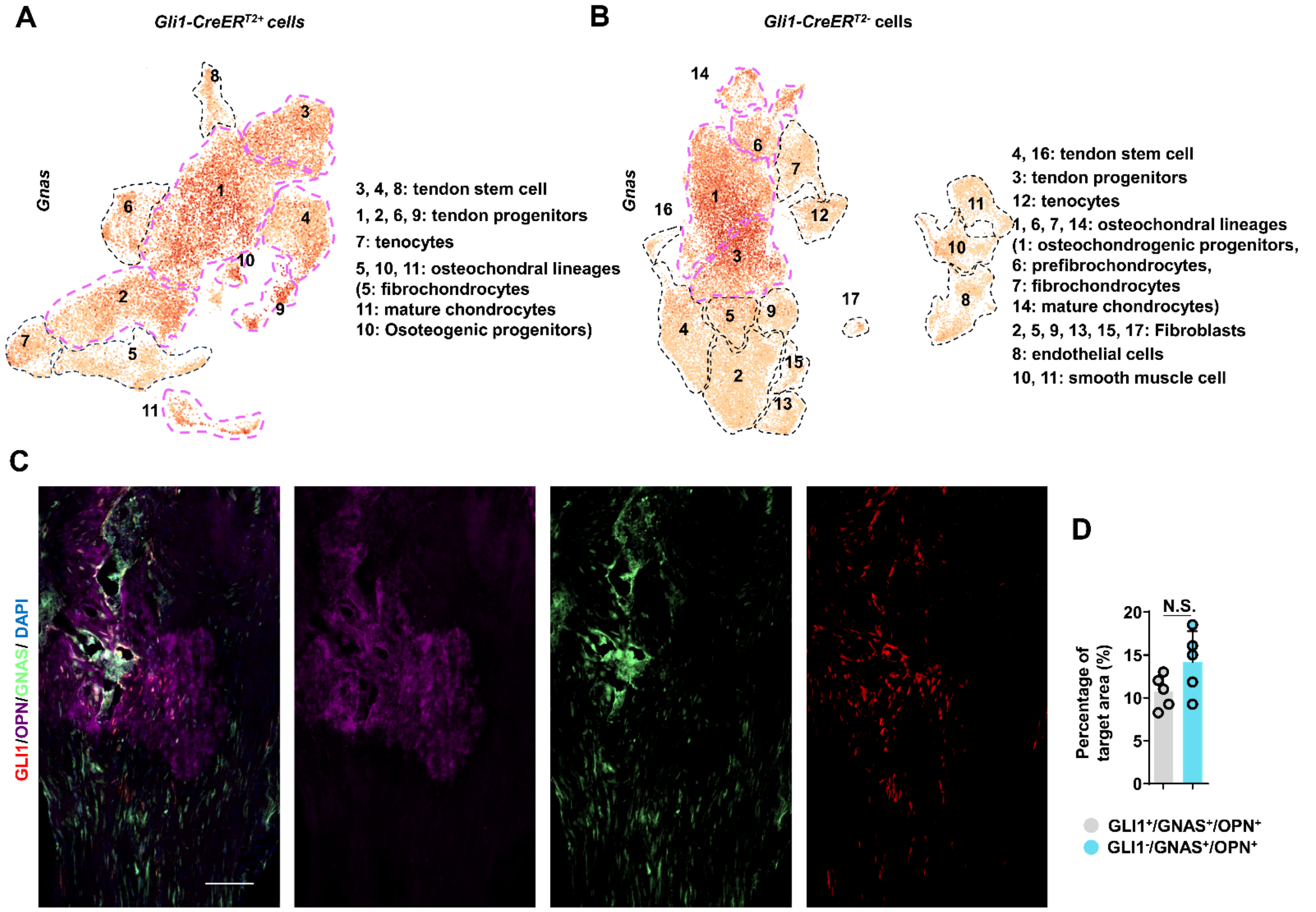

Appendix Figure S7. ***Gnas*$^{\mathbf{high}}$ cells represent osteochondrogenic progenitors and mature chondrocytes and osteoblasts.** (**A, B**) Feature plot images showing *Gnas* expression in *Gli1-CreER*$^{T2+}$ (**A**) and *Gli1-CreER*$^{T2-}$ (**B**) lineage cells. (**C, D**) Representative immunofluorescence (**C**) and statistical analysis (**D**) of GLI1, GNAS and OPN in injured site at 63 dpi (n=5 per group). Data is represented as the mean ± SD. N.S. indicated no significance. Scale bar, 100 μm.

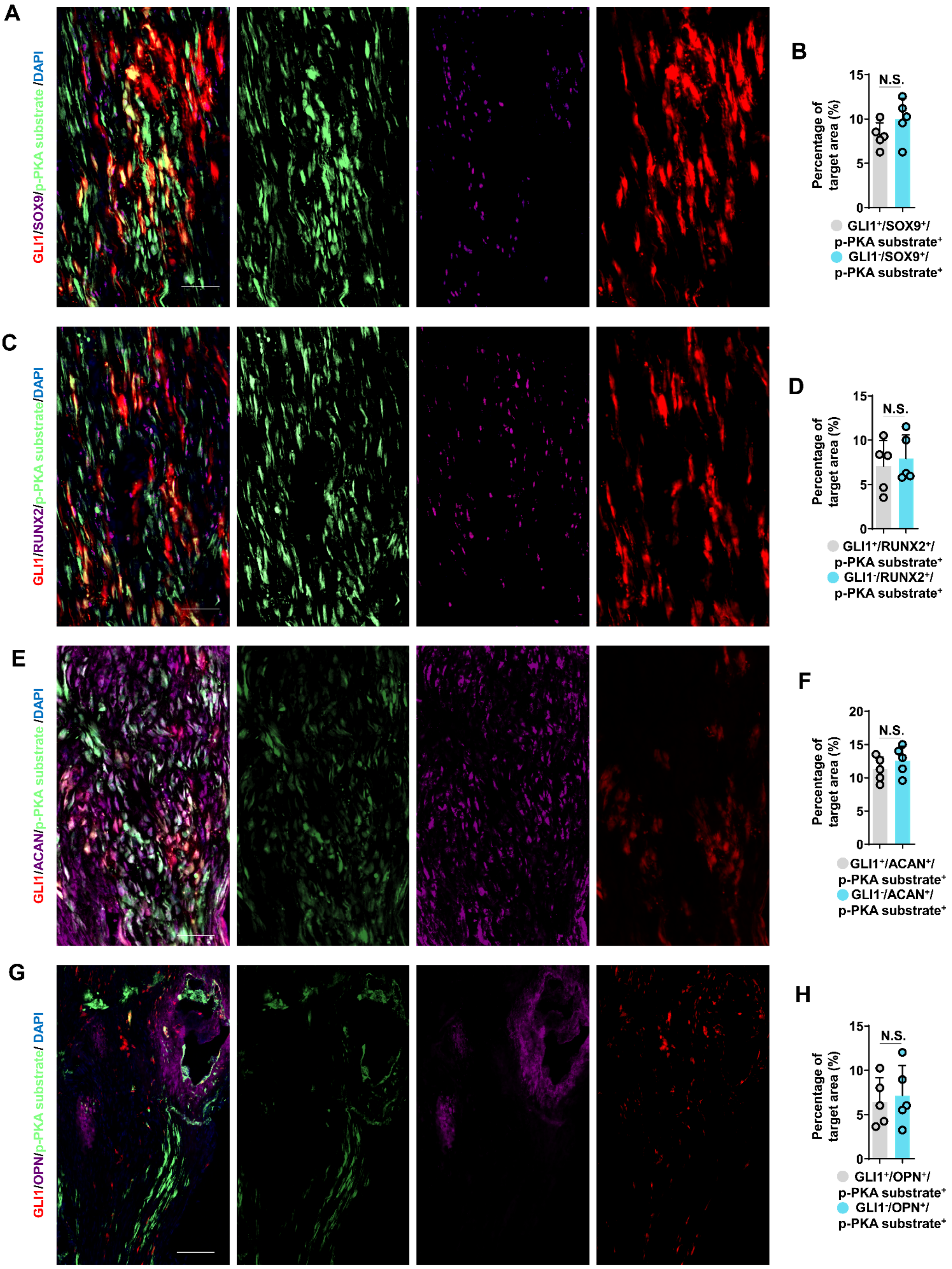

54

55 **Appendix Figure S8.** ***p*-PKA substrate^{high} cells represent osteochondrogenic progenitors and mature**

56 **chondrocytes and osteoblasts.** (**A, B**) Representative immunofluorescence (**A**) and statistical analysis (**B**) of

GLI1, *p*-PKA substrate and SOX9 in injured site at 7 dpi (n=5 per group). Data is represented as the mean ± SD. N.S. indicated no significance. Scale bar, 100 μm. (**C, D**) Representative immunofluorescence (**C**) and statistical analysis (**D**) of GLI1, *p*-PKA substrate and RUNX2 in injured site at 7 dpi (n=5 per group). Data is represented as the mean ± SD. N.S. indicated no significance. Scale bar, 100 μm. (**E, F**) Representative immunofluorescence (**E**) and statistical analysis (**F**) of GLI1, *p*-PKA substrate and ACAN in injured site at 21 dpi (n=5 per group). Data is represented as the mean ± SD. N.S. indicated no significance. Scale bar, 100 μm. (**G, H**) Representative immunofluorescence (**G**) and statistical analysis (**H**) of GLI1, *p*-PKA substrate and OPN in injured site at 63 dpi (n=5 per group). Data is represented as the mean ± SD. N.S. indicated no significance. Scale bar, 100 μm.

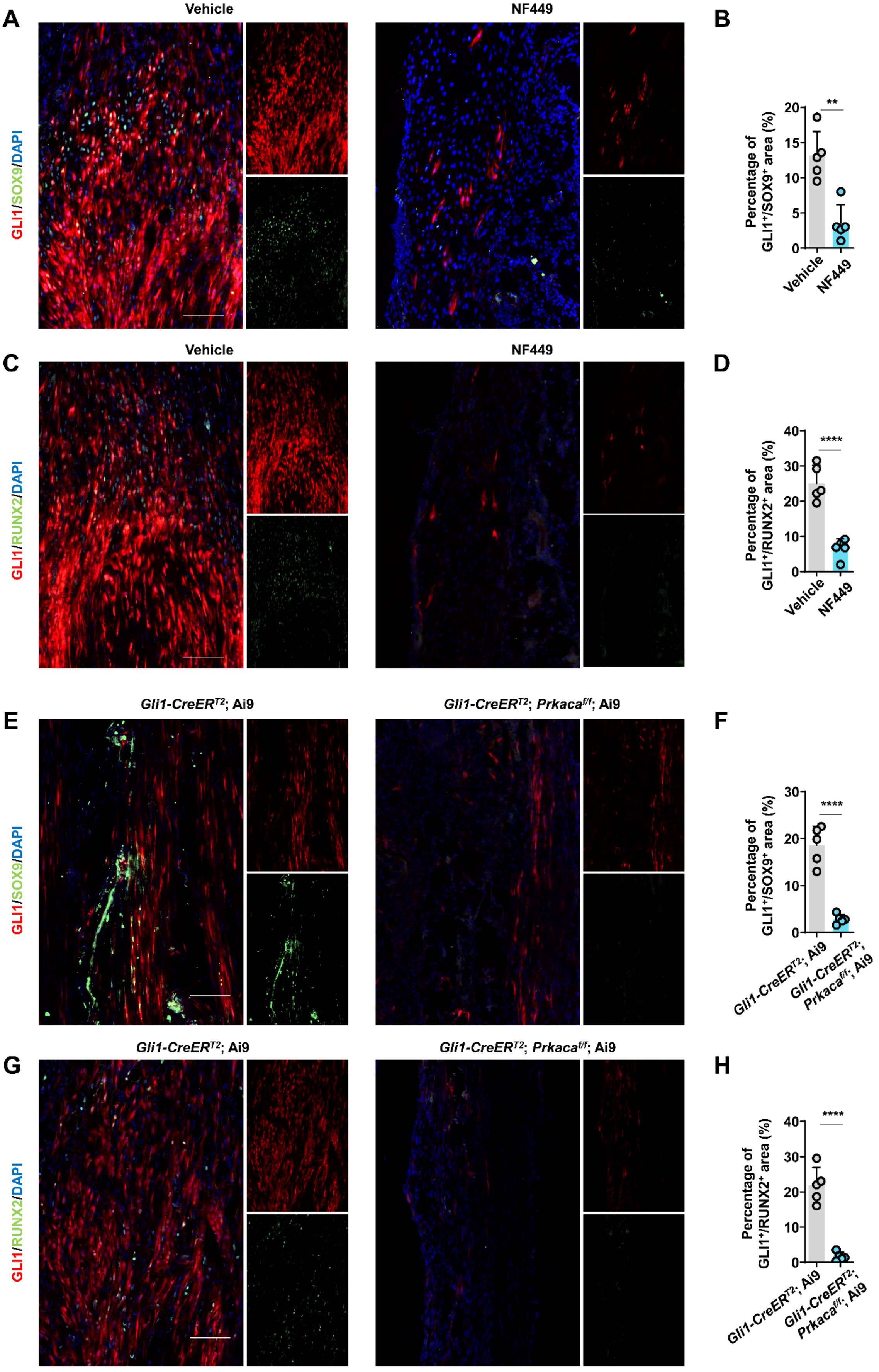

Appendix Figure S9. Pharmacological inhibition of Gαs and genetic PKA impairment disrupt osteochondral differentiation in *Gli1*⁺ tendon sheath progenitors. (A, B) Representative

immunofluorescence (**A**) and statistical analysis (**B**) of GLI1 and SOX9 in injured tendons of *Gli1-CreER*^{T2}; Ai9 mice with vehicle or NF449 treatment at 7 dpi (n=5 per group). ** $p < 0.01$. Scale bar, 100 μm. (**C, D**) Representative immunofluorescence (**C**) and statistical analysis (**D**) of GLI1 and RUNX2 in injured tendons of *Gli1-CreER*^{T2}; Ai9 mice with vehicle or NF449 treatment at 7 dpi (n=5 per group). **** $p < 0.0001$. Scale bar, 100 μm. (**E, F**) Representative immunofluorescence (**E**) and statistical analysis (**F**) of GLI1 and SOX9 in injured tendons of *Gli1-CreER*^{T2}; Ai9 and *Gli1-CreER*^{T2}; Ai9; *Prkaca*$^{f/f}$ mice at 7 dpi (n=5 per group). **** $p < 0.0001$. Scale bar, 100 μm. (**G, H**) Representative immunofluorescence (**G**) and statistical analysis (**H**) of GLI1 and RUNX2 in injured tendons of *Gli1-CreER*^{T2}; Ai9 and *Gli1-CreER*^{T2}; Ai9; *Prkaca*$^{f/f}$ mice at 7 dpi (n=5 per group). **** $p < 0.0001$. Scale bar, 100 μm. Data is represented as the mean ± SD. All p values were determined by unpaired Student's t test.

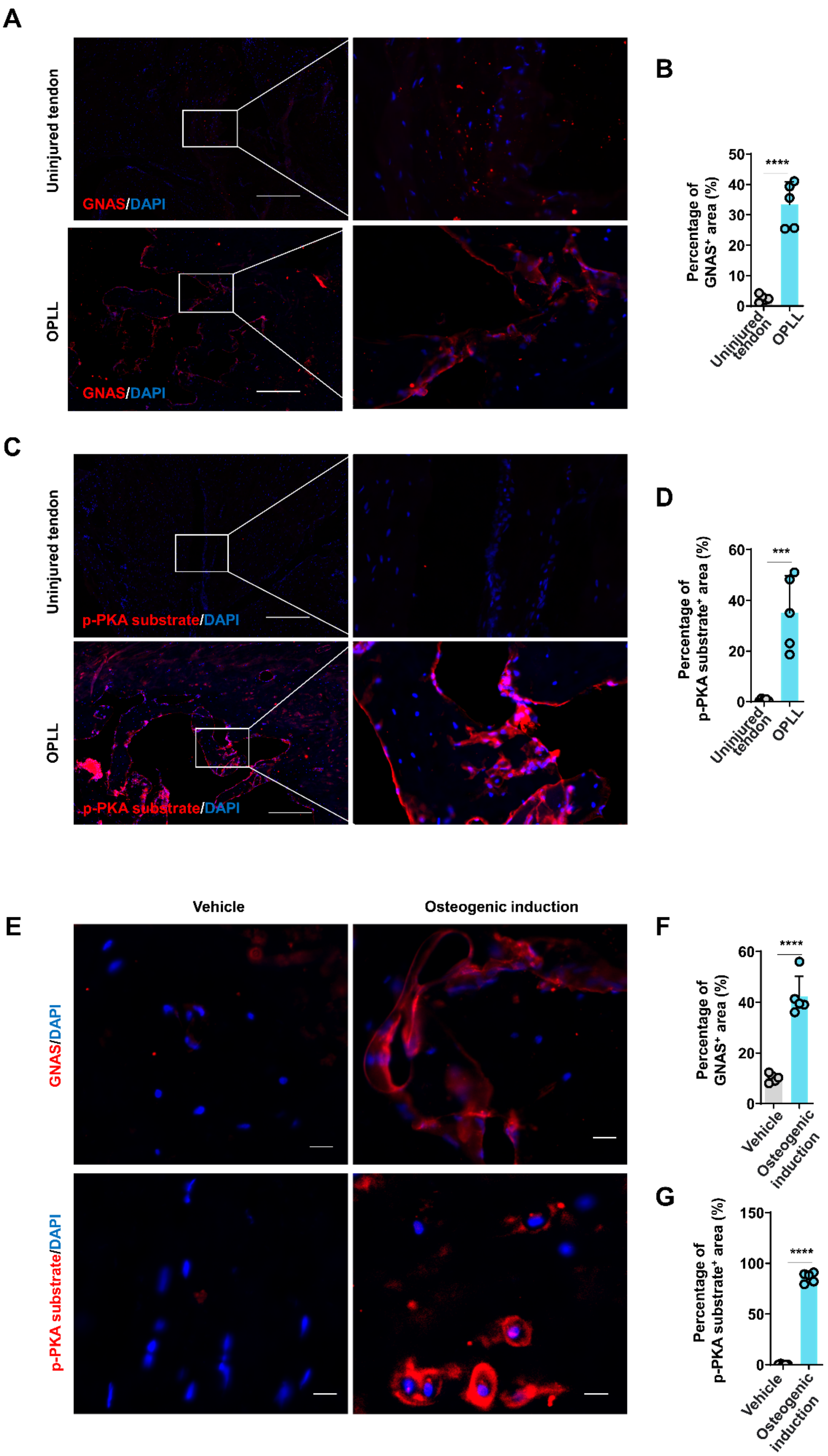

Appendix Figure S10. The frequency of GNAS$^+$ and *p*-PKA substrate$^+$ cells is higher in human HO lesions. (**A, B**) Representative immunofluorescence (**A**) and statistical analysis (**B**) of GNAS in normal tendon and HO lesion (n=3 per group). **** $p < 0.0001$. Scale bar, 100 µm. (**C, D**) Representative

immunofluorescence (**C**) and statistical analysis (**D**) of p-PKA substrate in normal tendon and HO lesion (n=3 per group). **** $p < 0.0001$. Scale bar, 100 μm. (**E**) Representative immunofluorescence staining for GNAS and p-PKA substrate in naïve (non-osteogenic control) and osteogenic tendon stem/progenitor cells. Scale bar, 10 μm. (**F, G**) Statistical analysis of GNAS (**F**) and p-PKA substrate (**G**) in naïve or osteogenic tendon stem/progenitor cells (n=5 per group). **** $p < 0.0001$. Data is represented as the mean ± SD. All *p* values were determined by unpaired Student's *t* test.
