# Supplementary material for: GNAS/PKA signaling promotes aberrant osteochondral differentiation of Gli1+ tendon sheath progenitors
Source: EMBO J. 2025 Sep 1;44(20):5890–917. doi: 10.1038/s44318-025-00553-7 (PMC12528478; doi:10.1038/s44318-025-00553-7)
Supplement: Supplementary file 4 — Expanded View Figures [file 44318_2025_553_MOESM4_ESM.pdf]

Expanded View Figures

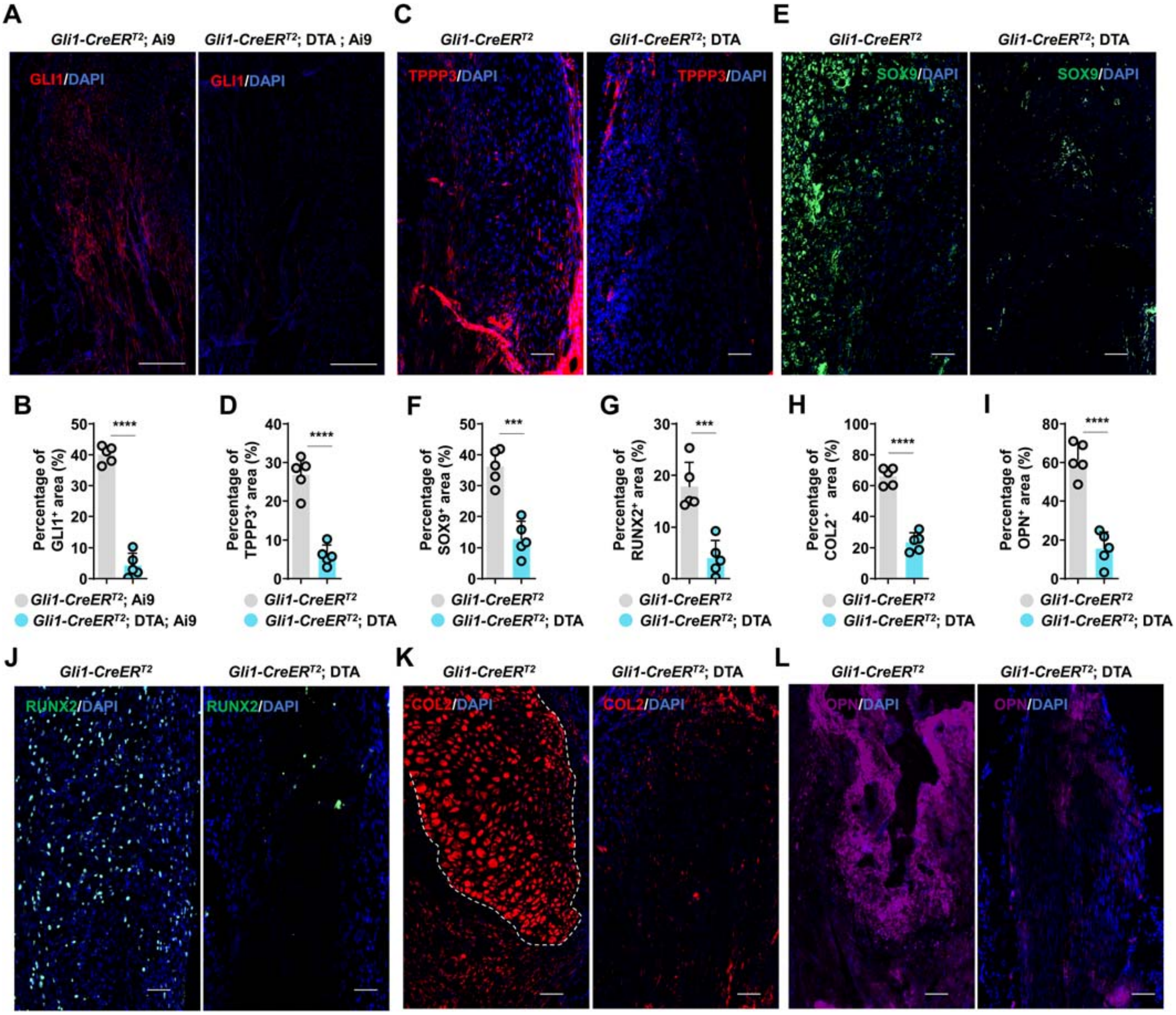

Figure EV1. ***Gli1*⁺ cell depletion significantly inhibited tendon stem/progenitor population and associated osteochondral differentiation.**

(**A**, **B**) Immunofluorescence staining and statistical analysis of the GLI1⁺ cells in injured tendons of *Gli1-CreER^{T2}*; Ai9 and *Gli1-CreER^{T2}*; Ai9; DTA mice at 21 dpi ($n = 5$ per group). ****$P = 1.74e-7$. Scale bar, 200 μm. (**C**, **D**) Immunofluorescence staining and statistical analysis of the TPPP3⁺ cells in injured tendons of *Gli1-CreER^{T2}* and *Gli1-CreER^{T2}*; DTA mice at 7 dpi ($n = 5$ per group). ****$P = 2.52e-5$. Scale bar, 200 μm. (**E**, **F**) Immunofluorescence staining and statistical analysis of the SOX9⁺ cells in injured tendons of *Gli1-CreER^{T2}* and *Gli1-CreER^{T2}*; DTA mice at 7 dpi ($n = 5$ per group). ***$P = 1.70e-4$. Scale bar, 200 μm. (**G–I**) Statistical analysis of the RUNX2⁺ (**G**), COL2⁺ (**H**) and OPN⁺ (**I**) cells in injured tendons of *Gli1-CreER^{T2}* and *Gli1-CreER^{T2}*; DTA mice at 7 dpi, 21 dpi and 63 dpi, respectively. ($n = 5$ per group). *P* values from left to right: ***$P = 7.22e-4$, ****$P = 3.24e-6$, ****$P = 3.54e-5$. (**J–L**) Immunofluorescence staining of the RUNX2⁺ (**J**), COL2⁺ (**K**) and OPN⁺ (**L**) cells in injured tendons of *Gli1-CreER^{T2}* and *Gli1-CreER^{T2}*; DTA mice at 7 dpi, 21 dpi and 63 dpi, respectively. Scale bar, 200 μm. Data is presented as mean ± SD. All *P* values were determined by unpaired Student's *t* test.

 © The Author(s)

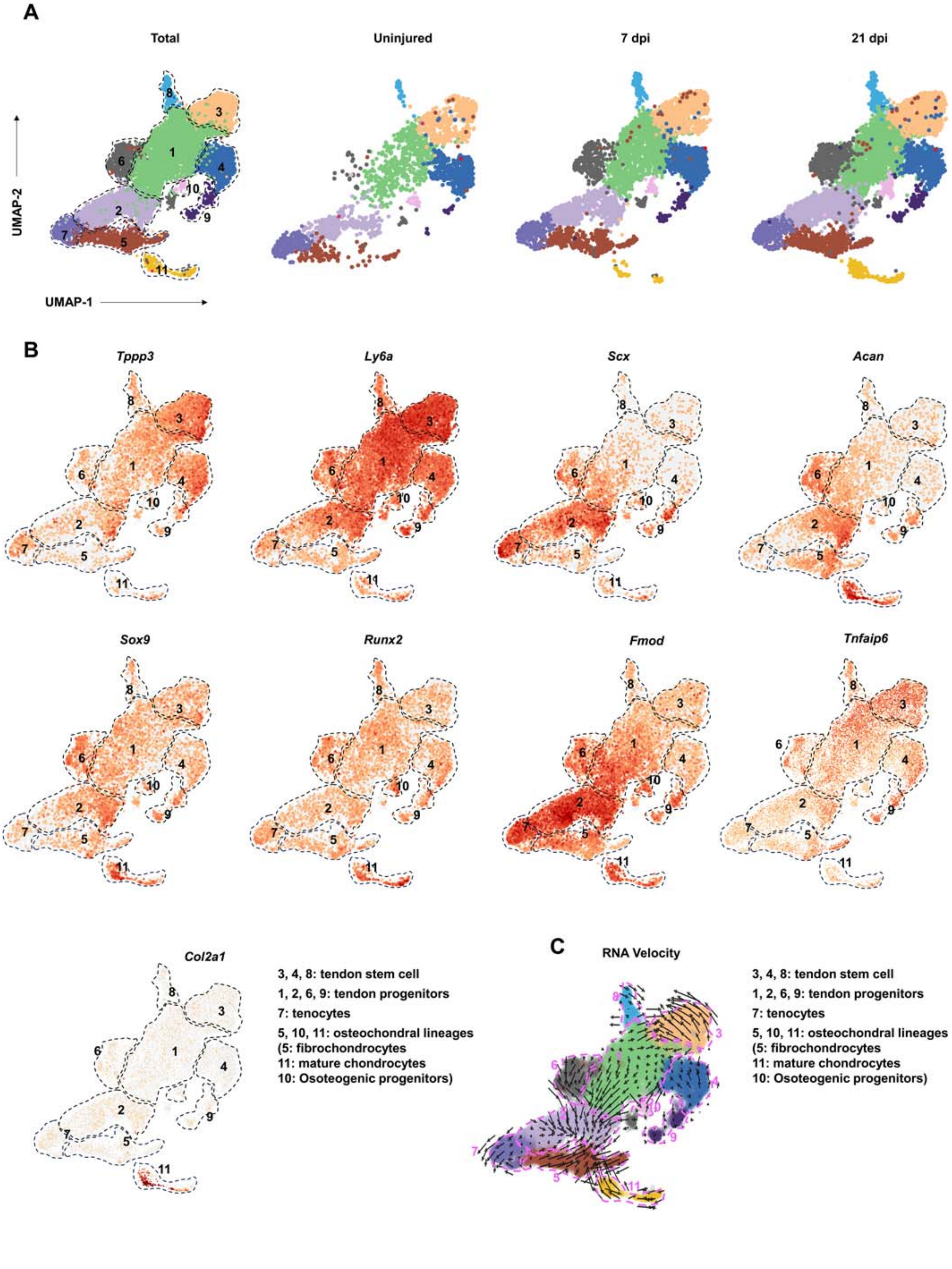
A
Total
Uninjured
7 dpi
21 dpi
UMAP-2
UMAP-1
B
Tppp3
Ly6a
Scx
Acan
Sox9
Runx2
Fmod
Tnfaip6
Col2a1
3, 4, 8: tendon stem cell
1, 2, 6, 9: tendon progenitors
7: tenocytes
5, 10, 11: osteochondral lineages
(5: fibrochondrocytes
11: mature chondrocytes
10: Osoteogenic progenitors)
C
RNA Velocity
3, 4, 8: tendon stem cell
1, 2, 6, 9: tendon progenitors
7: tenocytes
5, 10, 11: osteochondral lineages
(5: fibrochondrocytes
11: mature chondrocytes
10: Osoteogenic progenitors)

© The Author(s)

Figure EV2. *Gli1*$^+$ tendon sheath progenitors exhibited a multipotent capacity into tenogenic and osteochondrogenic lineages.

(**A**) UMAP visualization of *Gli1*$^+$ cells at uninjured and different time points post injury (i.e., 7 and 21 dpi). (**B**) Feature plot images showed the expression of recognized markers for tendon stem cells, progenitors and terminating tenocytes and chondrocytes. (**C**) RNA velocity showed the tenogenic and osteochondrogenic differentiation trajectory of *Gli1*$^+$ tendon sheath progenitors.

 © The Author(s)

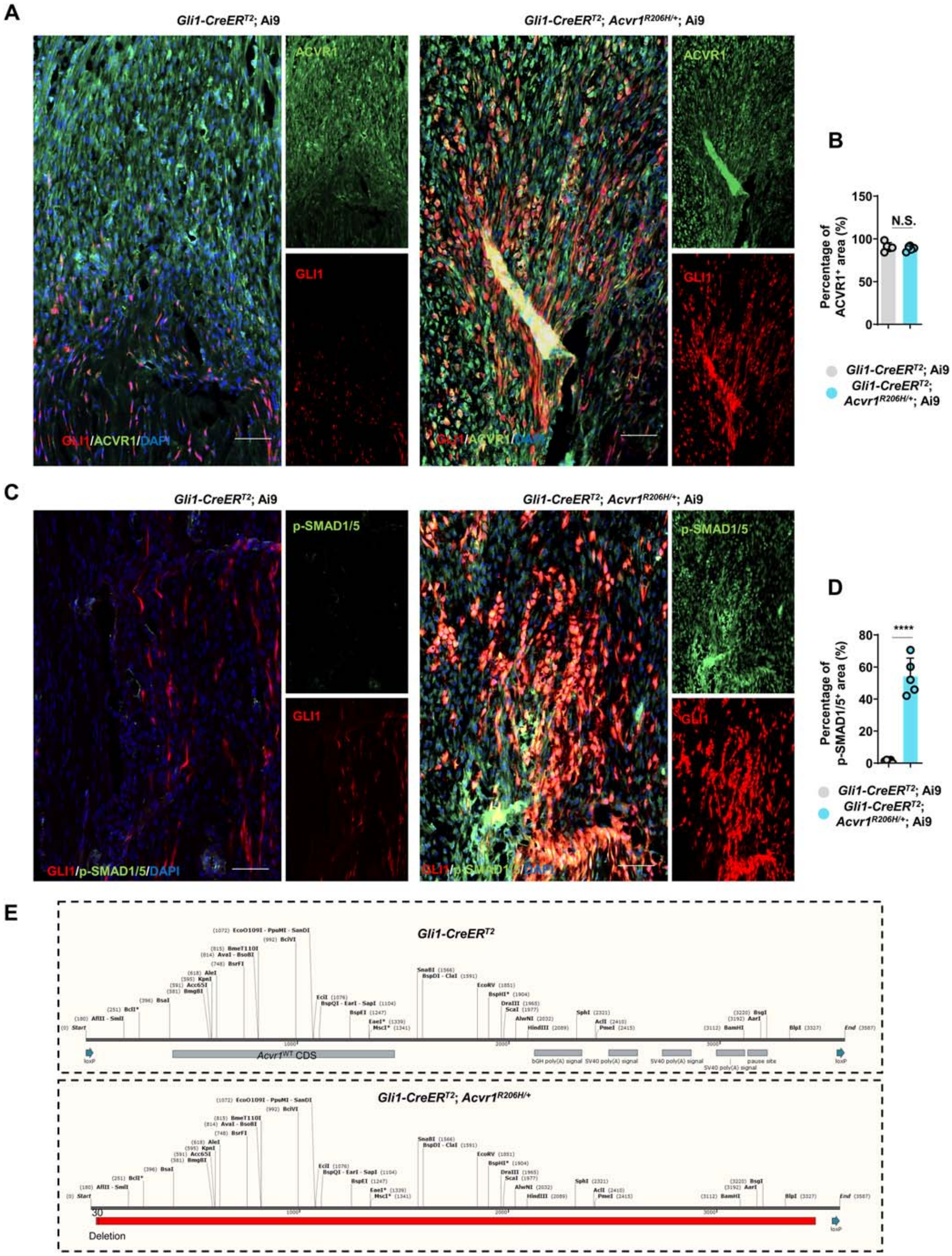
A
Gli1-CreERT2; Ai9
Gli1-CreERT2; Acvr1R206H/+; Ai9
ACVR1
GLI1
GLI1/ACVR1/DAPI
B
N.S.
Percentage of ACVR1+ area (%)
150
100
50
0
Gli1-CreERT2; Ai9
Gli1-CreERT2; Acvr1R206H/+; Ai9
C
p-SMAD1/5
GLI1/p-SMAD1/5/DAPI
D
****
Percentage of p-SMAD1/5+ area (%)
80
60
40
20
0
E
Gli1-CreERT2
Acvr1WT CDS
bGH poly(A) signal
SV40 poly(A) signal
pause site
loxP
Gli1-CreERT2; Acvr1R206H/+
Deletion

Figure EV3. Validation of *Acvr1* expression and *Acvr1*$^{R206H/+}$ mutation following *Gli1-CreER*^{T2} induction.

(**A**, **B**) Representative immunofluorescence and statistical analysis of GLI1 and ACVR1 in injured site of *Gli1-CreER*^{T2}; Ai9 and *Gli1-CreER*^{T2}; Ai9; *Acvr1*$^{R206H/+}$ mice at 5 dpi ($n = 5$ per group). $P = 5.18e\text{-}1$. N.S. indicated no significance. Scale bar, 100:µm. (**C**, **D**) Representative immunofluorescence and statistical analysis of GLI1 and p-SMAD1/5 in injured site of *Gli1-CreER*^{T2}; Ai9 and *Gli1-CreER*^{T2}; Ai9; *Acvr1*$^{R206H/+}$ mice at 5 dpi ($n = 5$ per group). ****$P = 6.89e\text{-}6$. Scale bar, 100 µm. (**E**) Sanger sequencing showed that the STOP cassette preceding the *Acvr1*$^{R206H/+}$ mutation was disrupted. Data is presented as mean ± SD. All *P* values were determined by unpaired Student's *t* test.

© The Author(s)

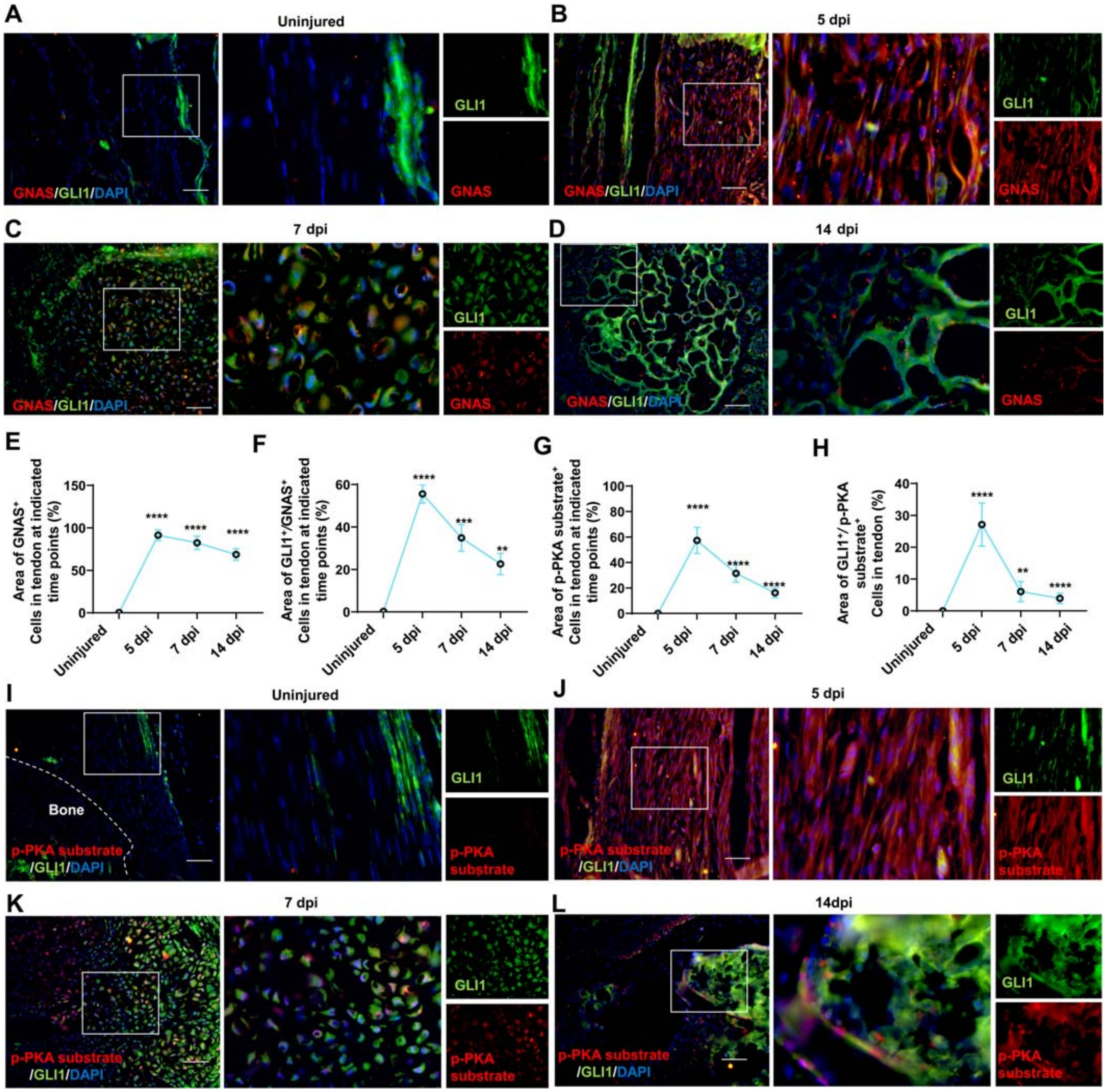

Figure EV4. GNAS/PKA signaling pathway was activated in osteochondral tendon progenitors of FOP mice.

(**A–D**) Representative immunofluorescence images of GNAS expression in uninjured tendons (**A**) and injured sites of *Gli1-CreER*^{T2}; Ai9 mice at 5 (**B**), 7 (**C**) and 14 (**D**) dpi. Scale bar, 50 µm. (**E**, **F**) Statistical analysis of GNAS$^+$ region (**E**) and GNAS$^+$/GLI1$^+$ region (**F**) in uninjured tendons and injured sites of *Gli1-CreER*^{T2}; *Acvr1*$^{R206H/+}$ mice at 5, 7 and 14 dpi ($n = 5$ per group). *P* values from left to right: ****$P = 8.29e-10$, ****$P = 1.16e-8$, ****$P = 1.57e-8$. ****$P = 2.36e-9$, ****$P = 1.71e-6$, ****$P = 9.06e-6$. (**G**, **H**) Statistical analysis of p-PKA substrate$^+$ region (**G**) and p-PKA substrate$^+$/GLI1$^+$ region (**H**) in uninjured tendons and injured sites of *Gli1-CreER*^{T2}; *Acvr1*$^{R206H/+}$ mice at 5, 7 and 14 dpi ($n = 5$ per group). *P* values from left to right: ****$P = 1.42e-6$, ****$P = 7.23e-6$, ****$P = 1.53e-5$. ****$P = 1.96e-5$, **$P = 2.63e-3$, ****$P = 6.44e-4$. (**I-L**) Representative immunofluorescence images of p-PKA substrate expression in uninjured tendons (**I**) and injured sites of *Gli1-CreER*^{T2}; *Acvr1*$^{R206H/+}$ mice at 5 (**J**), 7 (**K**) and 14 (**L**) dpi. Scale bar, 50 µm. Data is presented as mean ± SD. All *P* values were determined by unpaired Student's *t* test.

© The Author(s)

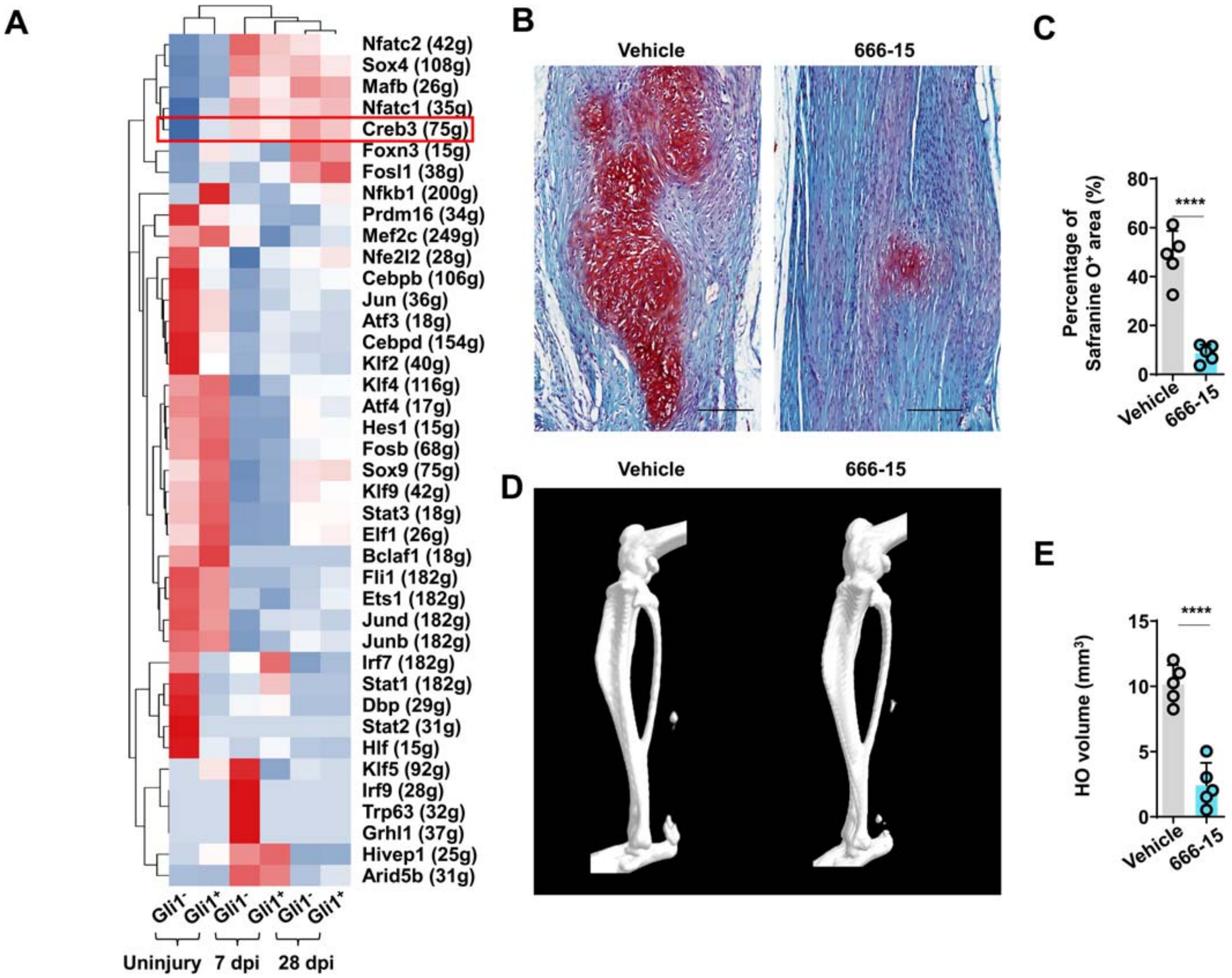

Figure EV5. 666-15, a CREB inhibitor, prevents tendon HO formation.

(**A**) Regulon analysis of transcription factors involved in the osteochondrogenic differentiation of *Gli1*+ and *Gli1*- cells. (**B**, **C**) Representative safranine O staining (**B**) and statistical analysis (**C**) of chondrocytes in injured site of tenotomized mice treated with vehicle or 666-15 treatment at 21 dpi ($n = 5$ per group). **$P = 4.50e-5$. Scale bar, 100 µm. (**D**, **E**) Representative microCT (**D**) and statistical analysis (**E**) of HO volume in injured site of tenotomized mice treated with vehicle or 666-15 treatment at 63 dpi ($n = 5$ per group). **$P = 5.93e-5$. Data is presented as mean ± SD. All P values were determined by unpaired Student's t test.

 © The Author(s)
